# Probabilistic Record Linkage for Families (PRLF): A Discussion of the Development and Validation of this Open-Source Linkage Tool

**DOI:** 10.23889/ijpds.v9i5.2763

**Published:** 2024-09-18

**Authors:** John Prindle, Himal Suthar, Emily Putnam-Hornstein, Regan Foust

**Affiliations:** 1 USC Suzanne Dworak-Peck School of Social Work; 2 UNC School of Social Work

Linking administrative records across programs can yield person-centered information, including client characteristics, public service trajectories, and outcomes and help to answer policy-related questions. Several solutions are available for undertaking record linkage, producing linkage keys for merging data sources for positively matched pairs of records. In this session, we will demonstrate a new application of the Python RecordLinkage package to family-based record linkages with machine learning algorithms for probability scoring, which we call probabilistic record linkage for families (PRLF). First, we will demonstrate the utility of PRLF with a simulation of administrative records and assess linkage accuracy with variations in match rates and data degradation. Second, we will compare generalized linear model estimates across three record linkage solutions (PRLF, ChoiceMaker, and Link Plus). Findings from the simulation study indicate linkage accuracy is largely influenced by degradation (e.g., missing data fields, erroneous or incomplete values) compared to the proportion of simulated matches between datasets. Results from the methods comparison using real world data indicate that all three solutions, when optimized, provide similar results for researchers. We discuss the strengths of our process, such as the use of ensemble methods, to improve match accuracy. We then will identify caveats of record linkage in the context of administrative data. The tool was developed in Python to allow for researchers to work with open-source software and adjust the basic workflow to fit their linkage needs. We will identify several partnerships where this collaboration has worked successfully and empower attendees with access to this useful tool.

